# Deep learning of echocardiography distinguishes between presence and absence of late gadolinium enhancement on cardiac magnetic resonance in patients with hypertrophic cardiomyopathy

**DOI:** 10.1186/s44156-024-00059-8

**Published:** 2024-10-14

**Authors:** Keitaro Akita, Kenya Kusunose, Akihiro Haga, Taisei Shimomura, Yoshitaka Kosaka, Katsunori Ishiyama, Kohei Hasegawa, Michael A. Fifer, Mathew S. Maurer, Yuichi J. Shimada

**Affiliations:** 1 Division of Cardiology, Department of Medicine, Columbia University Irving Medical Center, 622 West 168th Street, PH 3-342, New York, NY 10032 USA; 2 Department of Cardiovascular Medicine, Nephrology, and Neurology, Graduate School of Medicine, University of the Ryukyus, Okinawa, Japan; 3 Department of Medical Imaging Physics, Graduate School of Biomedical Sciences, Tokushima University, Tokushima, Japan; 4 Department of Cardiovascular Medicine, Tokushima University Hospital, Tokushima, Japan; 5 Department of Emergency Medicine, Massachusetts General Hospital, Harvard Medical School, Boston, MA USA; 6 Cardiology Division, Department of Medicine, Massachusetts General Hospital, Harvard Medical School, Boston, MA USA

**Keywords:** Hypertrophic cardiomyopathy, Echocardiography, Deep learning, Late gadolinium enhancement, Cardiac magnetic resonance

## Abstract

**Background:**

Hypertrophic cardiomyopathy (HCM) can cause myocardial fibrosis, which can be a substrate for fatal ventricular arrhythmias and subsequent sudden cardiac death. Although late gadolinium enhancement (LGE) on cardiac magnetic resonance (CMR) represents myocardial fibrosis and is associated with sudden cardiac death in patients with HCM, CMR is resource-intensive, can carry an economic burden, and is sometimes contraindicated. In this study for patients with HCM, we aimed to distinguish between patients with positive and negative LGE on CMR using deep learning of echocardiographic images.

**Methods:**

In the cross-sectional study of patients with HCM, we enrolled patients who underwent both echocardiography and CMR. The outcome was positive LGE on CMR. Among the 323 samples, we randomly selected 273 samples (training set) and employed deep convolutional neural network (DCNN) of echocardiographic 5-chamber view to discriminate positive LGE on CMR. We also developed a reference model using clinical parameters with significant differences between patients with positive and negative LGE. In the remaining 50 samples (test set), we compared the area under the receiver-operating-characteristic curve (AUC) between a combined model using the reference model plus the DCNN-derived probability and the reference model.

**Results:**

Among the 323 CMR studies, positive LGE was detected in 160 (50%). The reference model was constructed using the following 7 clinical parameters: family history of HCM, maximum left ventricular (LV) wall thickness, LV end-diastolic diameter, LV end-systolic volume, LV ejection fraction < 50%, left atrial diameter, and LV outflow tract pressure gradient at rest. The discriminant model combining the reference model with DCNN-derived probability significantly outperformed the reference model in the test set (AUC 0.86 [95% confidence interval 0.76–0.96] vs. 0.72 [0.57–0.86], *P* = 0.04). The sensitivity, specificity, positive predictive value, and negative predictive value of the combined model were 0.84, 0.76, 0.78, and 0.83, respectively.

**Conclusion:**

Compared to the reference model solely based on clinical parameters, our new model integrating the reference model and deep learning-based analysis of echocardiographic images demonstrated superiority in distinguishing LGE on CMR in patients with HCM. The novel deep learning-based method can be used as an assistive technology to facilitate the decision-making process of performing CMR with gadolinium enhancement.

**Supplementary Information:**

The online version contains supplementary material available at 10.1186/s44156-024-00059-8.

## Introduction

Hypertrophic cardiomyopathy (HCM) is one of the most common genetic cardiomyopathies [1]. The prevalence of clinically expressed HCM and genetic carrier of HCM is about 1 in 200 individuals [1]. Severe left ventricular (LV) hypertrophy, the main feature of this disease, can result in myocardial ischemia and subsequent fibrosis, which can be a substrate for fatal ventricular arrhythmia causing sudden cardiac death (SCD) [2–4]. 

Late gadolinium enhancement (LGE) on cardiac magnetic resonance imaging (CMR) typically represents myocardial fibrosis [5]. In patients with HCM, LGE is strongly associated with ventricular arrhythmia and subsequent SCD [6–9]. Appropriate use of implantable cardioverter-defibrillator (ICD) reduces disease-specific mortality [10–12]. The identification of subpopulation at high risk of SCD by LGE on CMR is important in HCM. However, not all patients with HCM undergo CMR as it is not widely accessible, is time-consuming, requires particular expertise for image acquisition and interpretation, and can be an economic burden for the patients and/or third-party payers [13]. Also, the risk-benefit balance needs to be carefully assessed in pediatric patients (who might require sedation or intubation) or those who have claustrophobia [14]. Furthermore, the use of some gadolinium enhancement agents is contraindicated in patients with end-stage renal disease [15–17]. For these reasons, it is clinically valuable to determine which patients with HCM would have a high pre-test probability of having LGE, thereby accelerating the appropriate use of CMR.

Deep learning is a rapidly evolving approach in a variety of medical settings including cardiovascular imaging [18–22]. This novel technology has the potential to overcome human limitations such as intra- and inter-observer variability [23, 24]. In the HCM population, previous studies demonstrated that deep learning-derived discrimination models using echocardiographic images can distinguish HCM from other cardiovascular diseases which cause LV hypertrophy [25–27]. Our recent study reported that deep convolutional neural network (DCNN) analysis of echocardiographic images can discriminate genotype positivity in patients with HCM [20]. However, despite the clinical importance, no previous studies examined the ability of deep learning to discriminate LGE on CMR in HCM. Therefore, we performed the present study to determine whether DCNN analysis of echocardiographic images can discriminate LGE on CMR in patients with HCM.

## Methods

### Study design and population

We conducted a cross-sectional study in HCM population between cases with LGE and controls without LGE. These patients were enrolled from the Columbia HCM Center at Columbia University Irving Medical Center (CUIMC) (New York, NY) between January 2008 and January 2022 and were consecutively included in this study if both transthoracic echocardiography (TTE) and CMR were performed. The diagnosis of HCM was established by echocardiographic evidence of LV hypertrophy (maximum LV wall thickness ≥ 15 mm) that was out of proportion to systemic loading conditions [2]. We excluded patients with HCM phenocopies such as Fabry disease, Danon disease, and cardiac amyloidosis by performing additional testing (e.g., genetic testing, technetium-99 m pyrophosphate scintigraphy imaging, and heart biopsy) when needed [2]. For patients with a family history of HCM, LV wall thickness ≥ 13 mm was considered diagnostic of HCM [2]. We excluded patients who underwent septal reduction therapy (i.e., septal myectomy, alcohol septal ablation –– interventions that may cause myocardial fibrosis) or heart transplant before initial TTE or CMR. We also excluded patients with suboptimal TTE images which were ineligible for DCNN analysis. The baseline characteristics were collected at the time of TTE which was performed closest to the date of CMR. The review board of CUIMC (AAAR5873) and Tokushima University Hospital (3217-5) approved the study protocol and all participants provided written informed consent to participate in the study before taking part. This study was developed following the Transparent reporting of a multivariable discriminant model for individual prognosis or diagnosis (TRIPOD) statement [28] and also the Proposed Requirements for Cardiovascular Imaging-Related Machine Learning Evaluation (PRIME) checklist (Table S1) [29]. 

### Outcome measure and acquisition of cardiac magnetic resonance images

The primary outcome was positive LGE on CMR. The existence but not extent of LGE was utilized as the primary outcome measure of the present study as LGE may affect the prognosis even if it is small [30] and also because there has been no consensus on an arbitrary cut-off percentage to define extensive LGE. Patients with LGE only located in the right ventricular insertion site were regarded as negative LGE, since the clinical significance or correlation with the prognosis of this finding has not been established [30–32]. CMR was ordered at the discretion of the treating physicians. CMR studies were performed on a 1.5-T field strength scanner (HDXt platform, General Electric Healthcare, Chicago, Illinois) with a dedicated 8-channel cardiac coil. The imaging protocol included localizer images with cine-balanced steady-state free precession imaging in the short axis, long axis, LV 4-chamber, and 3-chamber views. The myocardial late enhancement sequences were performed in LV short axis and 2-chamber views 8 to 15 min after the 0.2 mmol/kg injection of intravenous gadobutrol (Gadavist, Bayer HealthCare Pharmaceuticals Inc., Whippany, New Jersey). Short axis late enhancement views were obtained with both 2-dimensional single slice per breath-hold imaging and 3-dimensional volumetric ventricular imaging. Inversion times were determined on an individual basis to null the normal myocardial signal. The images were reviewed by expert readers using dedicated CMR analysis software (cmr^42^, Circle Cardiovascular Imaging Inc., Calgary, Alberta, Canada). Late myocardial enhancement images were analyzed using 2-dimensional views and coregistered 3-dimensional and long axis views for correlation when indicated [33]. The presence or absence of LGE was determined by the reading cardiac radiologist.

### Acquisition of the echocardiographic images

Standard TTE examinations were performed utilizing a commercially available ultrasound system (iE33, Philips Medical Systems, Andover, Massachusetts) as a part of routine clinical care in accordance with the guideline recommendations [34, 35]. The two-dimensional TTE images of all subjects were obtained from the apical 5-chamber views as this view can visualize most of the HCM-specific findings including LV outflow tract obstruction, interventricular septum, LV apex, and left atrium dilatation. The samples with good or adequate imaging quality on the basis of the visualization of the LV walls and endocardial borders were selected. TTE images were stored digitally as a Digital Imaging and Communication in Medicine (DICOM) file and analyzed offline.

### Import of the echocardiographic images

All DICOM images were cropped to 360 × 360 monochrome pixels and then down-sampled to 120 × 120 pixels. Simultaneously, metadata presented in the periphery of the images was removed. To adjust for differences in frame rate and heart rate between patients, 10 equally-spaced images per 1 cardiac cycle were chosen with the use of a semi-automatic heartbeat analysis algorithm. The starting frame was defined by the R wave on the electrocardiogram as recordings of TTE images were triggered by the R wave. The methodological details are provided in Supplemental Methods and have been published previously [18, 20]. 

### Deep learning algorithm

**Fig. 1:**
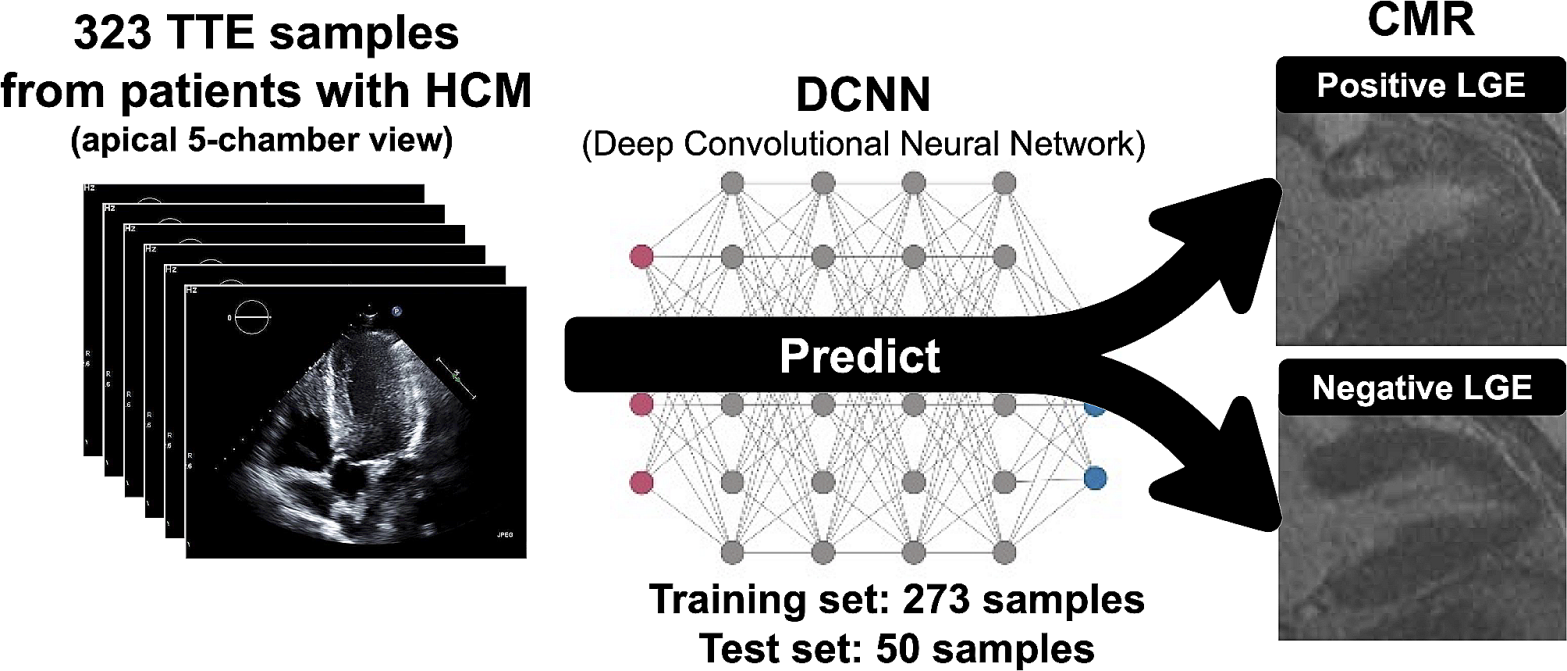
Graphical images of the deep convolutional neural network analysis. Using echocardiographic 5-chamber view images in patients with hypertrophic cardiomyopathy, the deep convolutional neural network-based discrimination model which differentiates positive and negative late gadolinium enhancement on cardiac magnetic resonance was developed. CMR, cardiac magnetic resonance; DCNN, deep convolutional neural network; HCM, hypertrophic cardiomyopathy; LGE, late gadolinium enhancement; TTE, transthoracic echocardiography

Positive LGE was discriminated by a DCNN algorithm using the apical 5-chamber view of each subject (Fig. 1). First, 50 samples were randomly selected as the independent test set and separated out. These 50 samples were not used for the model development. Second, the remaining 273 samples (the training set) were used for developing the discriminant model by performing 5-fold cross-validations within the training set (Figure S1). Model training was performed on a graphics processing unit (GeForce GTX 2080 Ti, NVIDIA, Santa Clara, California, USA) (Figure S2) [36]. The details are provided in Supplemental Methods. Deep learning was performed with the Python 3.6 programming language with Keras 2.2.4.

### Development of reference model and combining with DCNN-derived probability

In baseline characteristics, continuous variables were presented as mean ± standard deviation if normally distributed and as median [25th − 75th percentile] if not normally distributed. To compare the characteristics between patients with positive and negative LGE on CMR, the unpaired Student’s *t*-test was used for normally distributed continuous variables and the Mann-Whitney-Wilcoxon test for other continuous and ordinal variables. χ^2^ test was used for categorical variables.

As there were no existing models for discriminating positive and negative LGE in patients with HCM using clinical parameters, we developed a logistic regression model as a reference model to compare with the DCNN-derived model. The logistic regression model was developed using baseline characteristics with significant differences between patients with positive and negative LGE, including demographics, past medical history, family history, echocardiographic data, and CMR characteristics. This reference model was developed in the training set.

In the test set, the following steps were taken to compare the area under the receiver-operating-characteristic curve (AUC) of the reference model and that of a new model combining the reference model with the DCNN-derived model. First, a logistic regression model was constructed in the test set to estimate the correlation coefficients and the constant, combining the reference model and the DCNN-derived probability. Second, the AUC of the reference model and that of the combined model in the test set were compared using Delong’s test. The sensitivity, specificity, positive predictive value (PPV), and negative predictive value (NPV) in the test set were also calculated at the cut-off point with the best Youden index. Additionally, the calibration plot comparing the discriminative probability of the combined model and the actual prevalence of LGE on CMR in the test set was drawn. Decision curve analysis was also performed to examine how the combined model of the DCNN model plus the reference model could impact decision-making in the clinical settings. Statistical significance was declared if the 2-sided *P* value was < 0.05. For statistical analyses for baseline characteristics, developing the reference and combined model, and comparing the AUCs of them, R Studio version 2021.09.0 (Posit Software, Boston, Massachusetts) was utilized.

## Results

Among 340 CMR samples, 17 samples (5%) were excluded because the quality of TTE images obtained at the closest date to the CMR was inadequate for the DCNN analysis. A total of 323 CMR samples from patients with HCM — 160 positive LGE and 163 negative LGE — were included in the final analysis. The median time difference between TTE and CMR was 57 [25th − 75th percentile, 17–303] days. The training set comprised 273 samples, of which 135 (49%) had positive LGE. The independent test set comprised 50 samples, of which 25 (50%) had positive LGE.

Baseline characteristics are presented in Table 1. The proportion of patients with a family history of HCM was higher in the positive LGE group. In the echocardiographic parameters, LV end-diastolic diameter, maximum LV thickness, interventricular septal thickness, left atrium diameter, and the proportion of patients with LV ejection fraction < 50% were greater, and the peak pressure gradient via LV outflow tract was lower in the positive LGE group. In the CMR parameters, LV end-systolic volume was significantly greater in the positive LGE group. Therefore, the reference model included the following covariates: family history of HCM, maximum LV wall thickness, LV end-diastolic diameter on TTE, LV end-systolic volume in CMR, LV ejection fraction < 50%, left atrial diameter, and LV outflow tract pressure gradient at rest.

**Table 1:** Baseline clinical characteristics of the study sample

		Positive LGE (n = 160)	Negative LGE (n = 163)	*P* value
Demographics				
	Age (years)	54 ± 16	55 ± 18	0.50
	Female	56 (35)	75 (46)	0.06
	Race/Ethnicity			0.34
	European ancestry	79 (49)	92 (57)	
	African American	21 (13)	23 (14)	
	Asian	5 (3)	2 (1)	
	Native American	2 (1)	4 (3)	
	Unidentified	53 (33)	41 (25)	
	Height (cm)	170 ± 10	170 ± 12	0.51
	Weight (kg)	82 ± 19	83 ± 18	0.46
	BMI (kg/m^2^)	28 ± 6	29 ± 5	0.20
	Systolic blood pressure (mmHg)	127 ± 18	130 ± 21	0.12
	Diastolic blood pressure (mmHg)	72 ± 11	73 ± 11	0.42
Past medical history				
	NYHA functional class ≥ 3	29 (18)	28 (17)	0.94
	Hypertension	72 (45)	74 (45)	> 0.99
	Prior AF	34 (21)	22 (14)	0.09
	Prior sustained VT/VF	2 (1)	0 (0)	0.47
	Prior NSVT	26 (17)	19 (12)	0.27
	Prior syncope	28 (18)	30 (19)	0.95
Family history				
	Family history of SCD	20 (13)	24 (15)	0.67
	Family history of HCM	38 (24)	23 (14)	0.04
Medications				
	β-blocker	98 (62)	93 (57)	0.47
	Non-dihydropyridine calcium channel blocker	32 (20)	28 (17)	0.61
	Loop diuretic	14 (9)	16 (10)	0.89
	Aspirin	53 (33)	42 (26)	0.17
	Anticoagulation	26 (16)	15 (9)	0.08
	Thiazide	10 (6)	21 (13)	0.07
	ACE inhibitor	19 (12)	10 (6)	0.11
	ARB	13 (8)	28 (17)	0.02
	Potassium spearing diuretic	6 (4)	4 (3)	0.73
	Clonidine	0 (0)	6 (4)	0.04
	Statin	64 (40)	57 (35)	0.41
	Digoxin	2 (1)	1 (1)	0.98
	Disopyramide	10 (6)	1 (1)	0.01
	Amiodarone	5 (3)	2 (1)	0.43
Genetic testing (*n* = 155)		*n* = 84	*n* = 71	
	Pathogenic or likely pathogenic	37 (45)	21 (30)	0.08
Echocardiographic characteristics				
	LVDd (mm)	45 ± 6	43 ± 6	0.003
	LVDs (mm)	27 ± 7	26 ± 6	0.29
	Maximum wall thickness (mm)	18 ± 5	16 ± 5	0.002
	IVST (mm)	18 ± 5	16 ± 5	0.001
	LVPWT (mm)	12 ± 3	12 ± 3	0.90
	Left atrial diameter (mm)	44 ± 7	42 ± 6	0.02
	LV ejection fraction	64 ± 10	67 ± 5	0.002
	LV ejection fraction < 50%	12 (8)	1 (1)	0.004
	LV outflow tract gradient at rest (mmHg)	0 [0–28]	23 [0–55]	< 0.001
	LV outflow tract gradient with Valsalva (mmHg)	33 [0–54]	38 [0–77]	0.57
	Mitral valve SAM	59 (40)	73 (49)	0.33
	Degree of mitral regurgitation*	2.0 [1.0–2.5]	2.0 [1.0–2.5]	0.92
CMR characteristics				
	LV mass (g)	202 ± 64	180 ± 78	0.09
	LV end-diastolic volume (mL)	153 ± 39	148 ± 38	0.26
	LV end-systolic volume (mL)	61 ± 27	53 ± 20	0.004
	Stroke volume (mL)	92 ± 26	94 ± 23	0.60

Data are given as n (%), mean ± SD, or median [25th − 75th percentile]

^*^Degree of mitral regurgitation was converted to numerical values according to the following rule: none = 0, trace = 1, trace to mild = 1.5, mild = 2, mild to moderate = 2.5, moderate = 3, moderate to severe = 3.5, severe = 4

ACE, angiotensin-converting-enzyme; AF, atrial fibrillation; ARB, angiotensin II receptor blocker; ASA, alcohol septal ablation; BMI, body mass index; CMR, cardiac magnetic resonance; HCM, hypertrophic cardiomyopathy; IVST, interventricular septum thickness; LGE, late gadolinium enhancement; LVDd, left ventricular end-diastolic diameter; LVDs, left ventricular end-systolic diameter; LV, left ventricle; LVPWT, left ventricular posterior wall thickness; NSVT, non-sustained ventricular tachycardia; NYHA, New York Heart Association; SAM, systolic anterior motion; VT/VF, ventricular tachycardia or ventricular fibrillation

**Fig. 2:**
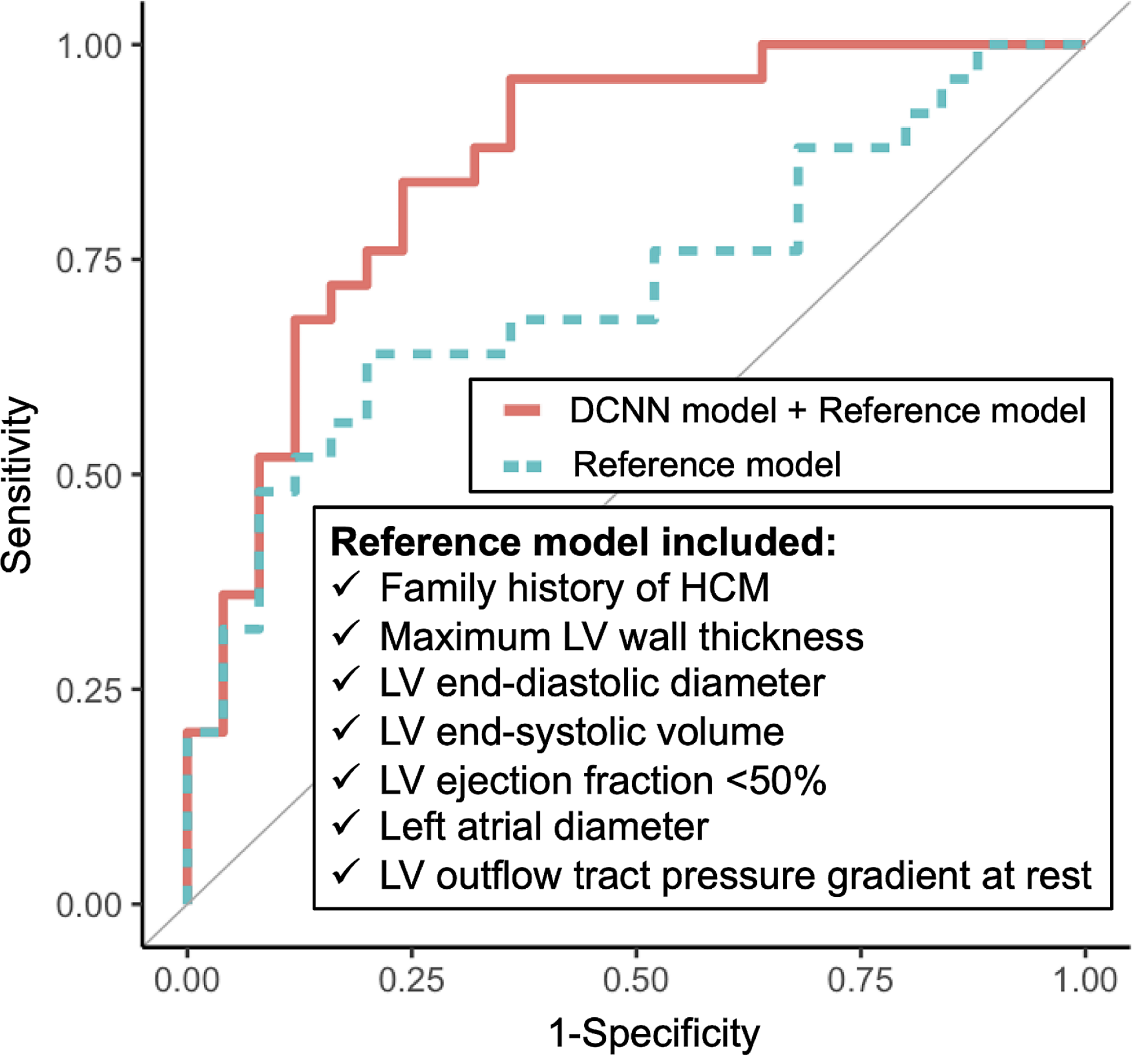
Comparison of receiver-operating-characteristic curves in the test set. To discriminate late gadolinium enhancement on cardiac magnetic resonance in patients with hypertrophic cardiomyopathy (HCM), the reference model (green dotted line) and the combined model (red solid line) were developed. The reference model included family history of HCM, maximum left ventricular (LV) wall thickness, LV end-diastolic diameter on echocardiography, LV end-systolic volume, LV ejection fraction < 50%, left atrial diameter, and LV outflow tract pressure gradient at rest. The combined model was developed by combining the reference model and deep convolutional neural network-based probability. DCNN, deep convolutional neural network; HCM, hypertrophic cardiomyopathy; LV, left ventricular

The AUC of the DCNN-derived probability of the discriminant model developed in the training set was 0.74 (95% confidence interval [CI] 0.60–0.88) in the independent test set. After combining the reference model with the DCNN-derived probability for discriminating positive LGE, the combined model significantly outperformed the reference model (AUC 0.86 [95% CI 0.76–0.96] vs. 0.72 [95% CI 0.57–0.86], Delong’s test *P* = 0.04) (Fig. 2). Table 2 summarizes the sensitivity, specificity, PPV, and NPV of each model. The correlation coefficients and constant for constructing the combined model are shown in Supplemental Results. The coefficients of each clinical parameter used in the reference model are shown in Table S2. The calibration plot in the test set is shown in Figure S3. The decision curve analysis (Figure S4) showed that the net benefit of the combined model was higher than the reference model in the clinically reasonable range of threshold probability.

**Table 2:** Comparison of the predictive performances between two models in the test set

Prediction model	AUC (95% CI)	*P* value*	Sensitivity† (95% CI)	Specificity† (95% CI)	PPV† (95% CI)	NPV† (95% CI)
Reference model	0.72 (0.57–0.86)	Reference	0.64 (0.43–0.82)	0.80 (0.59–0.94)	0.76 (0.53–0.92)	0.69 (0.49–0.85)
DCNN model + Reference model	0.86 (0.76–0.96)	0.04	0.84 (0.64–0.95)	0.76 (0.55–0.91)	0.78 (0.58–0.91)	0.83 (0.61–0.95)

^*^P value was calculated to compare the AUC of the reference model with that of the combined model, using Delong’s test

†The threshold probabilities of the reference model and the combined model to calculate each sensitivity, specificity, PPV, and NPV were 0.51 and 0.45, respectively, according to the cut-off points with the best Youden index

AUC, area under the receiver-operating-characteristic curve; CI, confidence interval; DCNN, deep convolutional neural network; NPV, negative predictive value; PPV, positive predictive value

## Discussion

### Summary of findings

In the present cross-sectional study of 160 cases with LGE and 163 cases without LGE among patients with HCM, the discriminative ability of the novel model combining the clinical-derived reference model and the DCNN-derived probability significantly outperformed that of the reference model in the independent test set. The present study serves as the first investigation demonstrating the additional value of deep learning-based analysis of echocardiographic images in discriminating LGE on CMR in patients with HCM.

### Clinical importance of discriminating LGE on CMR in HCM

LGE in HCM typically represents myocardial fibrosis [2–5]. The prevalence of LGE in the adult HCM population has been reported to be between 50 and 70%, which is in agreement with our finding (50%) [37]. Although LGE in HCM has been associated with an increased risk of SCD from ventricular arrhythmias [6–9], these potentially lethal arrhythmias can be appropriately aborted by ICD [10–12]. Identification of high-risk HCM subpopulations through detecting LGE on CMR contributes to reduced disease-specific mortality by subsequently facilitating ICD implantation [6–9]. However, in certain circumstances, patients have difficulties in undergoing CMR with gadolinium enhancement for SCD risk stratification due to accessibility, cost, MRI-incompatible implanted devices, and end-stage renal disease [13, 15–17]. Moreover, it is often challenging to perform MRI in pediatric patients and those with claustrophobia as they might require sedation or intubation [14]. Thus, it is clinically important to specify patients with HCM who have a high pre-test probability of LGE on CMR because CMR would be more likely to change their clinical management in such cases. Our new discrimination model based on deep learning analysis of echocardiographic images would help physicians and patients determine the utility of CMR more accurately. Furthermore, patients who cannot undergo CMR may benefit from this model of discriminating LGE as it would prompt considering alternative tests that can be implemented for further risk stratification (e.g., Holter monitoring) in high-risk patients.

### Clinical utility of the deep learning-based model in comparison with the reference model

In general, the threshold probability (x-axis) of the decision curve represents the minimum probability which would be required for a clinician and/or patient to order/undergo the management option of interest. In the present study, the management option of interest is CMR with gadolinium enhancement. The threshold probability is typically determined by balancing the perceived risk and benefit of the management of interest. In our study, the risk can be defined as complications of CMR with gadolinium enhancement such as claustrophobia event (0.7%) [14], allergic reaction (0.4%) [38], and nephrogenic systemic fibrosis (0.1%; prevalence of end-stage renal disease in HCM is 2–3% [39] and nephrogenic systemic fibrosis occurs in 3–7% after gadolinium enhancement in patients with end-stage renal disease, [17] thus the risk is ~ 0.1% in the overall HCM population), accounting for ~ 1.2% of overall patients with HCM. The benefit can also be defined as the prevention of SCD by ICD through accurate detection of LGE on CMR. Given that the prevalence of LGE was approximately 50–70% [37] and that 4.7% of patients with positive LGE experienced SCD, [9] the benefit can be roughly calculated as 2.4–3.3%. Assuming that CMR complications and SCD are equally important, the threshold probability of approximately 36–50% (= from 1.2/3.3 to 1.2/2.4) would be a reasonable estimation. In the decision curve analysis, our new model demonstrated greater net benefit than the reference model or performing CMR for all patients within and beyond this range of threshold probability, highlighting the clinical utility of our approach.

### Prior studies to discriminate LGE on CMR

The literature has documented various methods to predict LGE on CMR. A clinical model including a history of non-sustained ventricular tachycardia, reduced LV systolic function, and maximal echocardiographic LV wall thickness was able to discriminate extensive LGE [40]. However, the study excluded patients at high risk for SCD, limiting the generalizability [40]. Recently, two studies estimated the likelihood and extent of LGE based on electrocardiographic findings. The first study was relatively small (*n* = 42 including controls) and limited to patients who were 7–31 years old [41]. The second study used the Selvester QRS score to determine the presence and extent of LGE. Yet, it was limited by an extensive scoring system [42]. 

In addition to these clinical and electrocardiogram-based discriminant models, two studies developed discriminant models for LGE using CMR findings without gadolinium enhancement. One study utilized e-prime obtained from CMR for the discrimination of LGE [43]. Another study utilized balanced steady-state free precession cine sequences for the discrimination of LGE through deep learning algorithms [44]. Although these studies discriminated LGE based on CMR without gadolinium enhancement, they did not address the issue of limited accessibility, contraindications, and cost/resources associated with CMR itself.

These prior studies collectively underscore the clinical importance of discriminating LGE on CMR with other, less resource-intensive and more readily available, modalities. In this context, the ability of our deep learning-based approach to analyze echocardiographic images obtained in routine clinical care underscores the feasibility and generalizability of this novel method.

### Advantages of deep learning-based approach over the reference model

The reference model using the clinical parameters with significant differences between positive and negative LGE patients showed modest accuracy for the discrimination of LGE on CMR in the present study. In this reference model, 5 out of the 7 parameters were based on echocardiographic parameters, the measurement of which is occasionally interpreter-dependent, resulting in intra- and inter-observer variabilities [23, 24]. Additionally, even after going through specialized trainings, the interpretation of echocardiographic images can be subjective and affected by human fatigue. By contrast, deep learning has a potential to overcome such variability in the assessment of echocardiographic measurements by humans because deep learning-based models allow for an accurate, consistent, rapid, and automated interpretation of echocardiographic images while reducing the risk of human errors [45, 46]. Furthermore, the present DCNN approach not only utilizes spatial information but also encompasses temporal data by incorporating the additional dimension of time.

Deep learning algorithm has shown a high potential to revolutionize the process of diagnosis and prognostication in the fields of dermatology, [47] radiology, [48] and cardiology [18–20]. In the HCM population, a prior study reported that deep learning algorithms of echocardiographic images can distinguish HCM from cardiac amyloidosis and hypertensive LV hypertrophy [25–27]. The present study represents the first study to exhibit the additional value of deep learning-based analysis of echocardiographic images to discriminate positive LGE on CMR in patients with HCM.

### Potential limitations

The findings in the present study should be interpreted with several limitations in mind. First, the study sample consisted of patients who were enrolled in a tertiary care center and underwent CMR. Therefore, the inferences may not be generalizable to populations with less severe clinical manifestations or those who did not undergo CMR. Second, the sample size was relatively small for a study using DCCN, especially when the cohort was divided into the training and test sets. Third, a larger number of variables may show statistically significant differences and become included in the reference model if the sample size was larger. Fourth, LGE was treated as a binary variable (presence or absence) and quantification was not performed to identify the extent of LGE. Further investigations with larger sample sizes may enable us to estimate not only the presence but also the extent and location of LGE. Fifth, although extensive LGE is a class IIb recommendation for ICD implantation in the 2024 American guidelines, [2] this is not included in the European guidelines [3] and the presence or absence of LGE do not directly guide the decision of ICD implantation. Sixth, even if the pre-test probability for the presence of LGE is low, the patient should still undergo CMR when there are other appropriate indications, such as poor echocardiographic windows, the need to evaluate apical aneurysm/thrombus, and need for assessing myocardial perfusion. Seventh, the time difference between the CMR and the closest TTE was not the same, which might have resulted in disease progression in some patients. Eighth, not all patients with HCM underwent genetic testing. Ninth, myocardial samples were not available to confirm that the areas with LGE on CMR correspond with LV fibrosis. Last, by nature of the study design, no association with subsequent clinical outcomes such as SCD was evaluated.

## Conclusions

Compared to the reference model solely based on clinical parameters, our new model integrating the reference model and deep learning-based analysis of echocardiographic images demonstrated the superiority of distinguishing LGE on CMR in patients with HCM. For patients and treating physicians, the novel deep learning-based method in the present study can be used as an assistive technology to inform the decision-making process of performing CMR with gadolinium enhancement. These findings should also facilitate further investigations to specify which echocardiographic features the deep learning models are mainly utilizing to improve the discrimination of LGE on CMR in patients with HCM.

## Electronic supplementary material

Below is the link to the electronic supplementary material.

Supplementary Material 1

## Data Availability

No datasets were generated or analysed during the current study.

## Declarations

### Clinical trial number

This study does not have a clinical trial number as this is not a clinical trial.

## Abbreviations

CMR: Cardiac magnetic resonance imaging
DCNN: Deep convolutional neural network
HCM: Hypertrophic cardiomyopathy
ICD: Implantable cardioverter-defibrillator
LGE: Late gadolinium enhancement
LV: Left ventricle
SCD: Sudden cardiac death
TTE: Transthoracic echocardiography
