# Supplementary material for: Deep learning of echocardiography distinguishes between presence and absence of late gadolinium enhancement on cardiac magnetic resonance in patients with hypertrophic cardiomyopathy
Source: Echo Res Pract. 2024 Oct 14;11:23. doi: 10.1186/s44156-024-00059-8 (PMC11472433; doi:10.1186/s44156-024-00059-8)
Supplement: Supplementary file 1 — Supplementary Material 1 [file 44156_2024_59_MOESM1_ESM.pdf]

SUPPLEMENTAL MATERIALS

Deep Learning of Echocardiography Distinguishes between Presence and Absence of Late Gadolinium Enhancement on Cardiac Magnetic Resonance in Hypertrophic Cardiomyopathy

Keitaro Akita, MD, PhD, Kenya Kusunose, MD, PhD, Akihiro Haga, PhD, Taisei Shimomura, MS, Yoshitaka Kosaka, MS, Katsunori Ishiyama, BS, Kohei Hasegawa, MD, MPH, PhD, Michael A Fifer, MD, Mathew S Maurer, MD, Muredach P Reilly, MB, MSCE, and Yuichi J Shimada, MD, MPH

SUPPLEMENTAL METHODS

SUPPLEMENTAL RESULTS

SUPPLEMENTAL FIGURES

Figure S1. Details of the 5-fold cross-validation.

Figure S2. Structure of the deep convolutional neural network models.

Figure S3. Calibration plot of the combined model in the test set.

Figure S4. Decision curve analysis of the combined model and the reference model in the test set.

SUPPLEMENTAL TABLES

Table S1. Checklist of Proposed Requirements for Cardiovascular Imaging-Related Machine Learning Evaluation (PRIME).

Table S2. Details of the reference model.

SUPPLEMENTAL REFERENCES

SUPPLEMENTAL METHODS

Sample splitting and model development

Figure S1 illustrates the details of the 5-fold cross-validation of our deep convolutional neural network (DCNN) algorithm. A total of 50 samples were randomly selected as the independent test set. The remaining 273 samples were used as the training set to derive the discriminant model. These 273 samples in the training set were divided into a derivation fold (80%) and a validation fold (20%) to constitute one iteration. There were 5 iterations in each cross-validation cycle. In each one of the 5 iterations, the model parameters were optimized using 6 different learning rates. The cross-entropy error function was used to minimize the loss. The model with the minimal loss among the 6 learning rates was selected in each iteration. The model with the best performance of the 5 iterations was chosen as the best model of the cycle. The best model was used as the initial value of the next cycle of 5-fold cross-validation. This cycle was sequentially performed 10 times to enhance the model discriminant ability. Finally, the discriminant probability of the model was determined in each cardiac cycle in the independent test set. The probabilities for each cardiac cycle were finally averaged in each patient.

Classification model

Adam optimizer was used for training. **Figure S2** visualizes the DCNN model used in this study, where the network comprised a total of 5 convolutional layers of N, $2N$, $2N$, $2N$, and N filters with kernel sizes of 3×3×3 and 5 pooling layers of kernel size 2×2×2 were applied. A series of 2 fully connected layers – with 128 nodes and 5 units – were included in the final layer. Leaky Rectified Linear Unit (LeakyRelu) was used as an activation function in all activation layers except for the last activation, where the softmax function was employed. The number of

filters in the layers was controlled by N, and $N = 64$ was used in the present study. The models output a probability of positive LGE by 3D images (size of 120 pixels × 120 pixels × 10 images per cardiac cycle).

Data augmentation and retrieval

Each echocardiographic image was expanded and shrunk to yield 3 augmented images – i.e., the original, expanded, and shrunk. Then, each cardiac cycle was split by 10. In this process, the timing of the first R wave was regarded as time 0 and the second R wave as time 10, and image at equally split time 0, 1, 2, …and 9 were retrieved from each cardiac cycle. To adjust for differences in frame rate and heart rate, 10 equally spaced images per 1 cardiac cycle were selected with a semi-automatic heartbeat analysis algorithm.[1]

SUPPLEMENTAL RESULTS

For the logistic regression model combining the reference model with the DCNN-derived probability, the coefficient was 6.982 for the reference model and 7.471 for the DCNN-derived probability, and the constant was -7.594.

SUPPLEMENTAL FIGURES

Figure S1. Details of the 5-fold cross-validation.

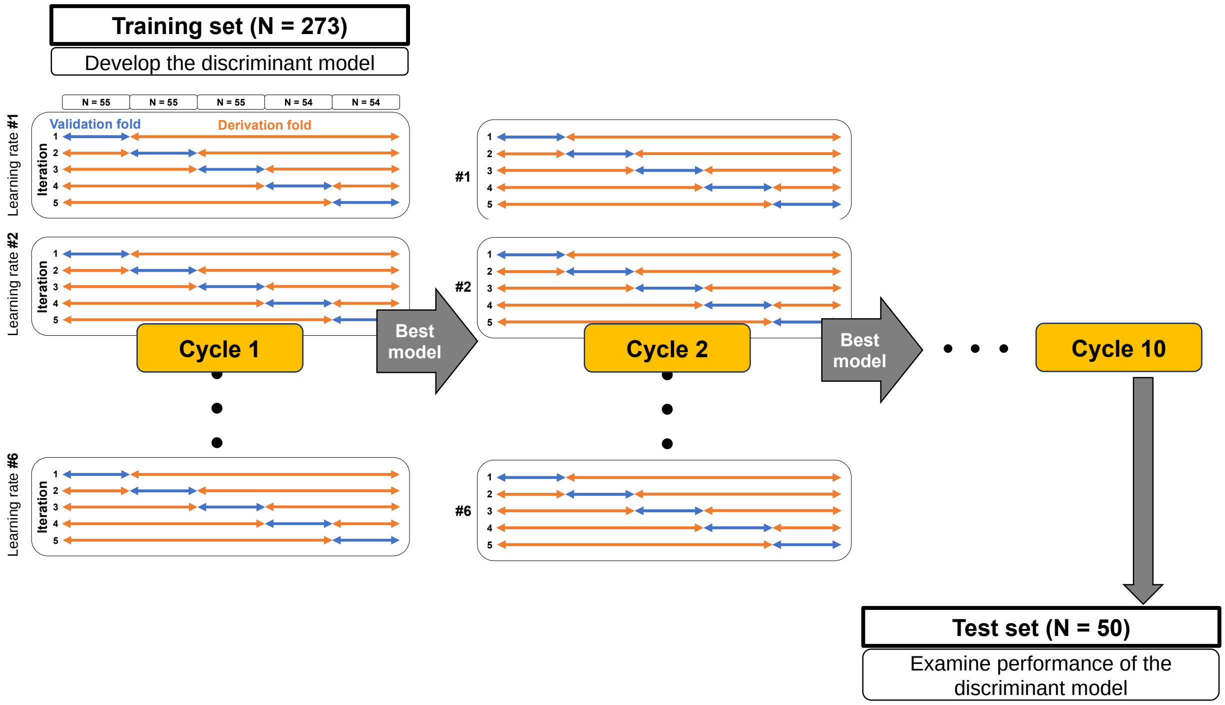

Figure S2. Structure of the deep convolutional neural network models.

DCNN, deep convolutional neural network.

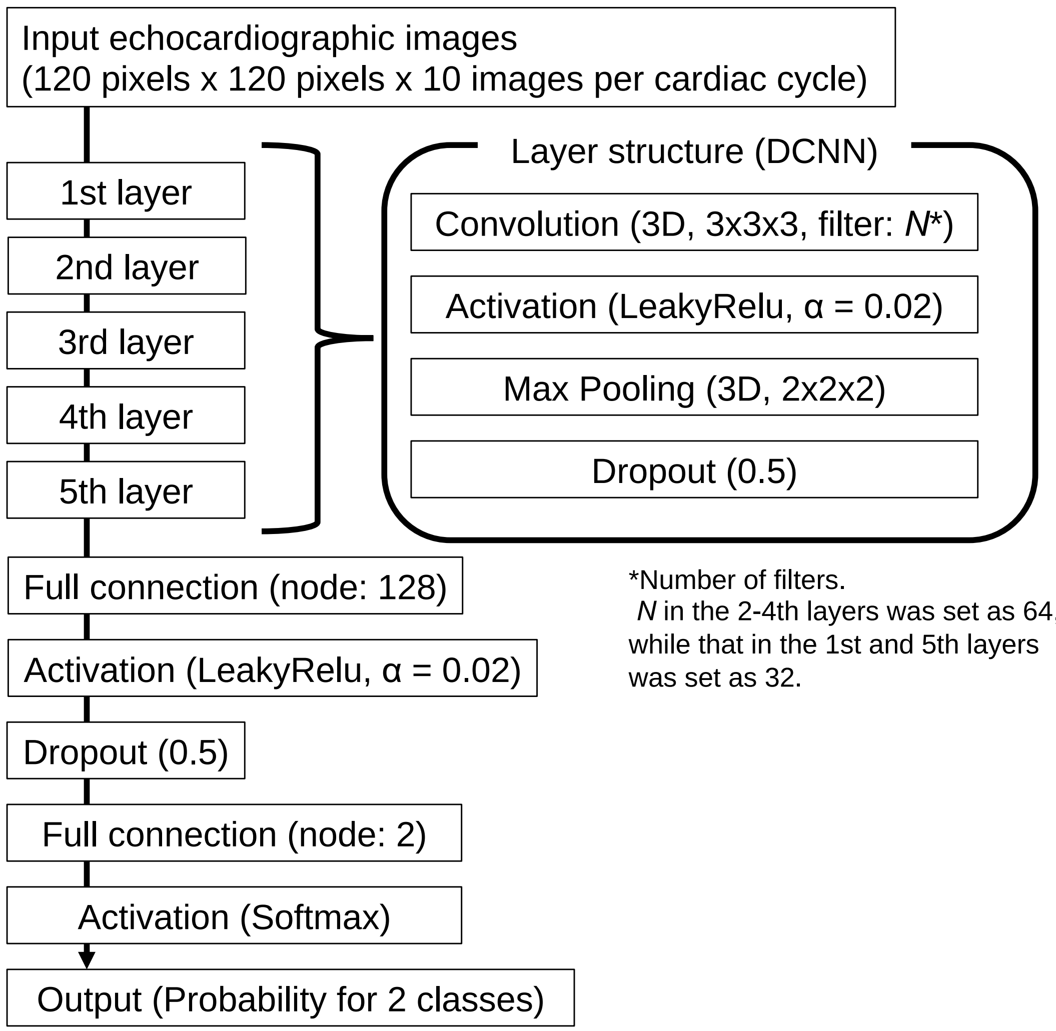

Figure S3. Calibration plot of the combined model in the test set.

The light blue area indicates the 95% confidence interval of the calibration plot.

LGE, late gadolinium enhancement.

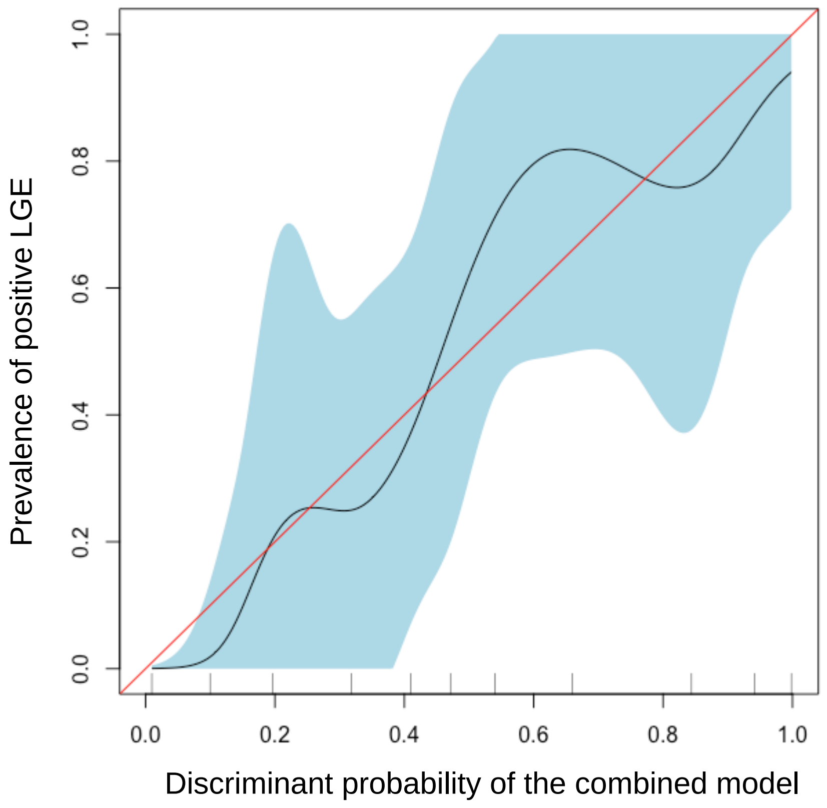

Figure S4. Decision curve analysis of the combined model and the reference model in the test set.

DCNN, deep convolutional neural network.

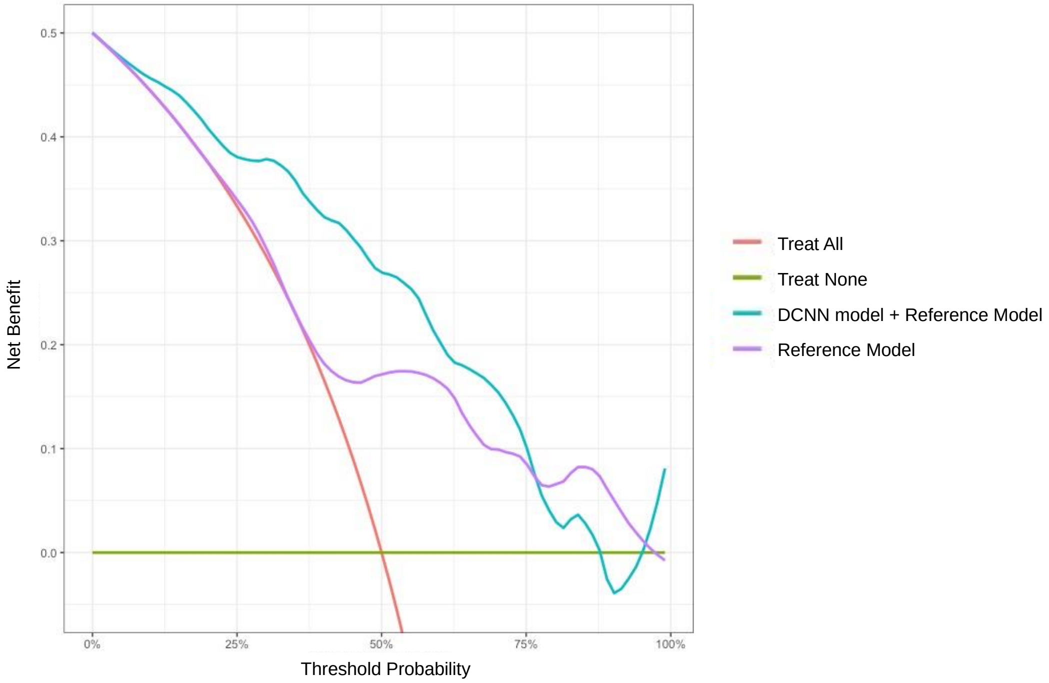

Table S1. Checklist of Proposed Requirements for Cardiovascular Imaging-Related Machine Learning Evaluation (PRIME).

Section	**Checklist item**	
1	**Designing the study plan**	
1.1	Describe the need for the application of machine learning to the dataset	pg 5
1.2	Describe the objectives of the machine learning analysis	pg 5
1.3	Define the study plan	pg 6, Fig 1
1.4	Describe the summary statistics of baseline data	pg 11
1.5	Describe the overall steps of machine learning workflow	pg 9-10, Fig 1, Fig S1-2
2	**Data standardization, feature engineering, and learning**	
2.1	Describe how the data were processed in order to make it clean, uniform, and consistent	pg 9-10, Fig 1, Fig S1-2, Supple Methods
2.2	Describe whether variables were normalized and if so, how this was done	N/A
2.3	Provide details on the fraction of missing values (if any) and imputation methods	pg 11
2.4	Perform and describe feature selection process	pg 10
2.5	Identify and describe the process to handle outliers if any	N/A
2.6	Describe whether class imbalance existed, and which method was applied to deal with it	N/A
3	**Selection of Machine Learning Model**	
3.1	Explicitly define the goal of the analysis e.g., regression, classification, clustering	pg 7
3.2	Identify the proper learning method used (e.g., supervised, reinforcement learning etc.) to address the problem	pg 9, Supple Methods
3.3	Provide explicit details on the use of simpler, complex, or ensemble models	pg 9-10, Supple Methods
3.4	Provide the comparison of complex models against simpler models if possible	pg 9-10
3.5	Define ensemble methods, if used	pg 9-10
3.6	Provide details on whether the model is interpretable	pg 11
4	**Model Assessment**	
4.1	Provide a clear description of data used for training, validation, and testing	pg 9, 11,12
4.2	Describe how the model parameters were optimized (e.g., optimization technique, number of model parameters etc.)	pg 9, Fig S1-2, Supple Methods
5	**Model Evaluation**	
5.1	Provide the metric(s) used to evaluate the performance of the model	pg 10
5.2	Define the prevalence of disease and the choice of the scoring rule used	pg 7
5.3	Report any methods used to balance the numbers of subjects in each class	N/A

5.4	Discuss the risk associated to misclassification	pg 16
6	**Best Practices for Model Replicability**	
6.1	Consider sharing code or scripts on public repository with appropriate copyright protection steps for further development and non-commercial use	pg 17
6.2	Release data dictionary with appropriate explanation of the variables	X
6.3	Document version of all software and external libraries	pg 9-10
7	**Reporting limitations, biases and alternatives**	
7.1	Identify and report the relevant model assumptions and findings	N/A
7.2	If well performing models were tested on a hold-out validation dataset, detail the data of that validation set with the same rigor as that of training dataset (see section 2 above)	pg 11-12

Table S2. Details of the reference model.

HCM, hypertrophic cardiomyopathy; LV, left ventricular.

**Variables**	**Coefficients**	**Standard error**	**Z value**	***P* value**
**Constant**	-5.37	1.44	-3.71	0.0002
**Family history of HCM**	0.27	0.34	0.80	0.42
**Maximum LV wall thickness**	1.27	0.34	3.78	0.0002
**LV end-diastolic diameter**	0.60	0.27	2.22	0.03
**LV end-systolic volume**	0.003	0.007	0.40	0.69
**LV ejection fraction <50%**	1.87	1.12	1.66	0.10
**Left atrial diameter**	0.16	0.24	0.65	0.52
**LV outflow tract pressure gradient at rest**	-0.01	0.005	-2.91	0.004

SUPPLEMENTAL REFERENCE

1. Kusunose K, Abe T, Haga A, Fukuda D, Yamada H, Harada M, et al. A deep learning approach for assessment of regional wall motion abnormality from echocardiographic images. JACC Cardiovasc Imaging. 2020;13:374-81.
